# Assessing the impact of multidrug-resistant tuberculosis in children: an exploratory qualitative study

**DOI:** 10.1186/1471-2334-14-426

**Published:** 2014-08-01

**Authors:** Caroline Franck, James A Seddon, Anneke C Hesseling, H Simon Schaaf, Donald Skinner, Lucy Reynolds

**Affiliations:** Faculty of Infectious and Tropical Diseases, London School of Hygiene and Tropical Medicine, London, UK; Desmond Tutu TB Centre, Department of Paediatrics and Child Health, Faculty of Medicine and Health Sciences, Stellenbosch University, Cape Town, South Africa; Department of Paediatric Infectious Diseases, Imperial College London, London, UK; Unit for Research on Health & Society, Faculty of Medicine and Health Sciences, Stellenbosch University, Cape Town, South Africa; Lady Davis Institute for Medical Research, Jewish General Hospital, 3755 Côte Ste-Catherine Road, Suite H-453, Montreal, H3T 1E2 QC, Canada

**Keywords:** South Africa, Childhood MDR-TB, Psychosocial impact, Qualitative research

## Abstract

**Background:**

While the prevalence of multidrug-resistant (MDR) tuberculosis (TB) is high among children in the Western Cape of South Africa, the psychosocial implications of treatment for children with MDR-TB remain poorly understood. We sought to explore how MDR-TB and its treatment impact children on an individual, familial, and social level.

**Methods:**

Semi-structured interviews were conducted with 20 children and caregivers purposively sampled from a prospective clinical cohort of children. The sample was stratified by age at the start of treatment (children >10 years, and 5-10 years). Caregiver proxy interviews were conducted with younger children, supplemented with child interviews; older children were interviewed directly, supplemented with caregiver proxy interviews. Data were analysed using grounded theory.

**Results:**

Findings revealed pill volume and adverse effects produced significant physical, psychological and academic disturbances in children. Adverse effects related to the medication were important obstacles to treatment adherence. While there appear to be no long-lasting effects in younger children, a few older children showed evidence of persisting internalised stigma. Caregivers suffered important treatment-related financial and psychological costs. Community support, notably through the continued involvement of children in strong social networks, promoted resilience among children and their families.

**Conclusions:**

We found that the current treatment regimen for childhood MDR-TB has significant psychological, academic, and financial impacts on children and their families. There is a need for psychosocial support of children and caregivers to mitigate the negative effects of community stigma, and to manage the stressors associated with chronic illness.

**Electronic supplementary material:**

The online version of this article (doi:10.1186/1471-2334-14-426) contains supplementary material, which is available to authorized users.

## Background

Drug-resistant tuberculosis (TB) was referred to as a public health crisis in the 2013 World Health Organization (WHO) Global TB Report. An estimated 450,000 people developed multidrug-resistant (MDR)-TB (i.e. caused by strains resistant to at least isoniazid and rifampicin) in 2012 [1], with childhood estimates as high as 32,000 in 2010 [2]. The treatment of childhood MDR-TB can be challenging: typically, children are hospitalised when injectable agents are administered (usually for the first 4 to 6 months), and continue outpatient treatment for a further 12 to 18 months [3–6]. Child-friendly drug formulations remain scarce and medications are frequently unpalatable, producing numerous adverse effects including nausea, vomiting and diarrhoea [6, 7]. However, the psychosocial impact of MDR-TB and its treatment remain virtually unstudied, and little is known about how children experience long-term hospital admission, medication, and medicalisation resulting from the disease.

A recent collection of children’s stories from the global MDR-TB community described a number of psychosocial challenges faced by these children worldwide, including persisting stigma, abandonment, mental illness, and lack of financial, emotional and parental support [8]. As a result, we sought to obtain information concerning children’s individual, familial, and social experiences of MDR-TB, with the aim of uncovering the impact of disease and treatment on their lives. To our knowledge, ours is the first study to use rigorous scientific tools to record, extract, analyse, and examine the experiences of children following treatment for MDR-TB. We anticipate that these study findings will provide a framework for future interventions in the management of childhood MDR-TB.

## Methods

### Setting and study population

This study took place in the Western Cape province of South Africa, one of the top MDR-TB high-burden settings worldwide [1]. Provincial government estimates from 2013 indicated between 450 and 600 incident cases of MDR-TB (adult and children) are diagnosed in this setting each year [9]. More generally, recent studies have documented an increase in MDR-TB in South Africa, including an increase in confirmed disease among children [10, 11]. Health outcomes for these children are variable: a 2011 study indicated that 53% of 968 South African children and adolescents with drug-resistant TB were cured or completed treatment, while 24% died [12]. However, other reports show more than 90% cure or treatment completion [13].

The Western Cape province is comprised of two dominant ethnic groups: Black African and Cape Coloured (mixed race), who remain largely racially segregated across neighbourhoods and townships [14]. The study sample frame was comprised of a prospective cohort of 148 children referred to the Tygerberg Children’s Hospital and Khayelitsha outreach clinics who were started on treatment for MDR-TB between 1^st^ January, 2009 and 31^st^ December, 2010 [13]. Twenty children were purposively sampled from this frame to obtain variation in ethnicity, age and gender. Developmental considerations restricted the sampling frame to children at least 5 years of age at the start of MDR-TB treatment. Other exclusion criteria included: children residing over one hour’s drive from the research facility, families who were untraceable, or children or caregivers who were not available for interviews.

### Data collection

Primary caregivers were contacted by a TB counsellor to obtain verbal consent allowing the child to take part in the study. The study rationale and objectives were explained to caregivers in their native language (isiXhosa/Afrikaans). To minimise the methodological challenges of interviewing children [15–17], participants were classified into two broad age strata. Older children, at least 10 years of age at the start of treatment, were interviewed directly, supplemented thereafter with caregiver interviews. Younger children, between five and ten years of age at the start of treatment, were interviewed by caregiver proxy, and subsequently directly interviewed. Interviews took place in participants’ home or in the privacy of the researcher’s vehicle. All interviews were assisted by a research counsellor acting as an interpreter for isiXhosa or Afrikaans-speaking participants, the two dominant local languages. Questions followed a semi-structured topic guide on the experience of diagnosis and treatment, with specific discussion following participants’ narrative lead. Open data collection facilitated the emergence of themes put forth in participants’ own narratives, and the subsequent generation of theory grounded in their accounts. The interpreter immediately translated participant responses for the interviewer, who could speak neither language. All original isiXhosa and Afrikaans interview recordings were later translated and transcribed by an independent translator so as to retain the highest degree of response accuracy. English recordings were transcribed by the first author. Data collection was terminated following evidence of approaching data saturation.

### Data analysis

Transcribed interview data were analysed by the first author, using principles of grounded theory [18]. Coded categorical themes were internally validated by comparative analysis, whereby emerging themes were compared with similar trends in the same and different interviews. Memos were written to synthesise ideas and uncover thematic relationships across categories and between interviews. This process facilitated the recording of stages of analytical development, serving as a platform upon which to interpret the raw data and develop a comprehensive theory. Interviews were subsequently re-read for evidence of findings negating our theory. Finally, analyses were cross-checked with co-authors and colleagues familiar with the study context. This manuscript complies with the RATS guidelines for reporting qualitative research [19].

### Ethical considerations

Signed informed consent was obtained from the parent/legal guardian (caregiver) for their interview, as well as on behalf of the child. The child’s written assent was obtained separately, where appropriate. Participants were assured of confidentiality and anonymity in written work. All voice files, transcripts, and consent/assent forms were safely stored in the research facilities. This study was approved by the Human Research Ethics Committee of Stellenbosch University, Western Cape, South Africa, and the Ethics Committee of the London School of Hygiene and Tropical Medicine, London, UK.

## Results

A total of 36 children were identified who met the eligibility criteria, from which 20 children were purposively sampled (Additional file 1). The demographic characteristics of the children interviewed are summarised in Table 1. Of the caregivers contacted who declined to participate, reasons included unavailability during the study period, or time conflicts due to employment.

**Table 1:** **Demographic characteristics of study participants**

Participant age & identifier*	Sex	Ethnicity	HIV status	Duration of hospital admission
**Younger children (age 5-10 years at start of MDR-TB treatment)**				
7a	F	Black	Negative	3 months, 6 days
7b	F	Black	Positive	NA
8	F	Coloured	Negative	5 months, 21 days
9a	F	Black	Positive	7 months, 5 days
9b	F	Coloured	Negative	NA
10	F	Black	Positive	5 months, 9 days
11a	F	Coloured	Negative	NA
11b	F	Black	Negative	5 months, 7 days
11c	F	Coloured	Positive	13 months, 4 days
11d	F	Coloured	Negative	4 months, 1 day
11e	F	Black	Negative	4 months
12	F	Coloured	Negative	3 months, 18 days
**Older children (age ≥10 years at start of MDR-TB treatment)**				
14a	M	Black	Positive	3 months, 24 days
14b	M	Black	Negative	6 months, 28 days
15a	M	Black	Positive	19 months, 25 days
15b	M	Black	Positive	4 months, 27 days
15c	M	Coloured	Negative	2 months^†^
17a	F	Coloured	Positive	8 months, 3 days
17b	F	Coloured	Negative	1 month, 18 days
17c	M	Black	Positive	1 month, 28 days

Abbreviations: *F* Female, *HIV* Human immunodeficiency virus, *M* Male, *MDR* Multidrug-resistant tuberculosis, *NA* Not admitted, received outpatient treatment.

*Unique identifiers were assigned to all participants. For example: “7a” refers to Child age 7, participant “a.” Ages are reported at the time of interview.

†Self-reported: admission/discharge dates unavailable.

Three broad themes were identified in participants’ narratives, including I) Problems with the clinical course of treatment, II) Relationships with the self, and III) Relationships with others. Themes and subthemes are presented and discussed within this framework.

### Problems with the clinical course of treatment

#### Medications: adherence and adverse effects

The majority of respondents cited the large number of pills and their adverse effects to be the most challenging components of treatment.

*The lot of pills. For a child, the pills are too big to swallow.* (Caregiver, 11a)

*They make me feel dizzy. […] And they made me feel pain in my stomach.* (Child, 11b)

*Oh, those drugs it is not for a child to use, […] because a child’s system can’t hold them.* (Caregiver, 11c)

*Sometimes when I smell the pills, I just feel like I must vomit.* (Child, 11e)

Adverse effects coupled with exceptionally long treatment duration rendered adherence difficult, particularly among HIV-positive children concurrently taking anti-retroviral therapy (ART) and treatment for MDR-TB.

*You know, [my son] is having lots of pills now. HIV pills and MDR, it’s too much. Even his MDR treatment is more than mine.* (Caregiver, 14a)

*[During 4 months] I was hiding my pills. They were not nice.* (Child, 15b)

*I thought to myself, ‘my medication is becoming more and more, couldn’t it be less?’ Because the more it became, the lazier I became in taking them. I know they are going to make me better, but I’m just tired of [pills] because I have been taking [ART] from a young age, as young as 3 years old until now.* (Child, 17a)

*I was not taking my pills. […] They were bitter and they made me vomit.* (Child, 17c)

The challenges of medical treatment were also felt by caregivers, who reported frequent struggles to obtain children’s cooperation:

*“It was a fight when it came to the pills, every day.”* (Caregiver, 11a)

#### Academic impact

Children had access to in-hospital schooling during their admission to Brooklyn Chest or Tygerberg hospitals. When asked what they found most challenging following their return to a normal academic institution, a few children cited difficulties related to prolonged absenteeism.

*They have classes [at Brooklyn Chest Hospital (BCH)], but it’s not the same as normal school. I didn’t get used to the longer days, because at BCH classes were 2 hours. At school it’s much longer, so that was hard. The workload changed as well. But other than that it was fine. (Child, 17b)*

*[What was most difficult was] when I started reading again, because I couldn’t read Afrikaans [anymore]. (Child, 12)*

Caregivers cited the challenges of children attending school when experiencing adverse effects from the medication.

*Those tablets were making him very, very dizzy. I was worried in the morning when he must go to school, you can see he is dizzy.* (Caregiver, 14a)

*When the schools were closed she could eat her treatment and then relax a bit, but there was no time for that when she had to go to school. Those were the times she would vomit.”* (Caregiver, 11e)

Children who were made to repeat the same academic grade found it easier to avoid questions in a new peer group, whereas those who joined their old classmates in the next grade faced more intense questioning and speculation.

*When she went back to school, everyone was pointing now. “There’s the girl that’s been out of school for 6 months.”* (Caregiver, 11a)

*The hardest part about going back to school was that my friends were not going to ask me where I was and why I was out of school for so long. If I had to tell them the truth then their attitude towards me would change. (Child, 17a)*

A few caregivers used the term “slow” to describe a change in the child’s academic abilities, or even more generally, their intellectual capabilities.

*[A]fter that treatment she forgets everything. […] I’m worried because when she did grade one before she went to hospital, she was so clever, very, very brilliant. (Caregiver, 9a)*

*She is a bit slow, she is not doing well at school anymore so next year she is going to another school. I think [her old friends] have noticed she is different, because she does not play with the big children, she plays with the small children. (Caregiver, 11c)*

*The school was calling me, she was very slow at her work. (Caregiver, 11d)*

While no cause was identified for this trend, decreased academic performance and behavioural changes may be attributed to a range of factors, including adverse effects from medications, and the difficulty of returning to the pace of a normal academic curriculum.

#### Financial impact

The financial impact of treatment on families was a dominant theme. The most frequently cited expenditures included travel to hospital and food-related expenses.

*Sometimes it would be hard in terms of food, and then I would not be able to give her the pills […] because the pills would just sit in the stomach.* (Caregiver, 7b)

*It was very difficult for us when she got TB because we can’t give her the food we eat, you see. […] Sometimes the bread must have peanut butter, it must have jam, must have a little bit of cheese—I can’t, I haven’t got money. (Caregiver, 10)*

*We didn’t plan on this thing and now it’s out of our budget. It’s taxi fare, and it’s staying in hospital…* (Caregiver, 11a)

Treatment-related expenditures were frequently compounded with a loss of family income.

*At the time I found out about [my child’s] condition I was working, but then I lost my job while I was looking after her in the hospital.* (Caregiver, 7a)

*When she was [in hospital] it was so suffering. […] Because I wasn’t working, my daughter was not working, then we were travelling with the transport.* (Caregiver, 11b)

*The doctor also booked me off [of work] because [I also had TB and] I might infect other people.* (Caregiver, 14a)

Some respondents cited government grant payments as helpful in offsetting the financial costs of treatment, whereas others believed them to be insufficient.

*When [the child was in hospital], the social worker came to talk to me so I told her I went to cash loans to borrow money, so I have to bring that money back when I get my grant. […] Everything has gone back to normal [now].* (Caregiver, 9a)

*It’s only [my] husband that is working. […] One child each gets R280, that is the “All Pay” [government grant], and then it is the husband’s salary. Then it is all the expenses that we have, you see.* (Caregiver, 8)

*[T]here came a time when we had to re-apply for [the child’s] grant. Then [the] money was finished, so then my mother helped me.* (Caregiver, 14b)

While a minority of respondents claimed they had been deemed ineligible for grant payments, it was not clear to them why these had been denied.

### Relationships with the self

#### Impact on self-perception

As evidenced from their ambitious future career aspirations, most children experienced no negative treatment-related impact on their sense of perceived self-efficacy.

*[Fashion] modelling.* (Child, 11b)

*I will finish school, then I will go to college and after college I want to work.* (Child, 11d)

*I want to play soccer.* (Child, 14a)

*I want to be a doctor. […] I saw that doctors help people, they even helped me when I didn’t know if I was going to live.* (Child, 15a)

When asked whether TB had changed them, or their life, in any way, most respondents reported no lasting change.

*No, nothing [has changed].* (Child, 14a)

*Not much has changed actually. I used to swim a lot. I don’t swim as often but I still like swimming and it doesn’t feel different. I run out of breath more easily now, but other than that everything is fine.* (Child, 17b)

Respondents evidenced little self-blame for having contracted MDR-TB.

*How can [my son] not get TB because of the way he is [child is cognitively impaired] and the place we are living in?* (Caregiver, 14b)

*I just accepted it. […] I asked myself who I got it from […] but then I would answer myself by saying that maybe I got it here because the place is dirty.* (Child, 15b)

*I was sad because I’d started school, obviously when I went back I would have lots of work to catch up on, but other than that, nothing. […] I didn’t feel like it was my fault.* (Child, 17b)

A minority of respondents acknowledged a persisting negative change in demeanour following TB treatment.

*I would say that my attitude has changed because when I had completed my treatment I was rude to everyone. […] I’m alone most of the time, I only have this one friend who also has TB, I just decided to befriend her.* (Child, 17a)

*He changed now, when he came back from [the hospital]. […] He is now a difficult child.* (Caregiver, 17c)

Overall, older children and adolescents appeared to have internalised their condition to a greater extent than did younger children.

### Relationships with others

#### Disclosure of disease

Disclosure of MDR-TB status revealed four dominant themes: selective, open, secondary, and no disclosure. Most respondents expressed selective disclosure attitudes.

*I told [some friends] that I have something in the air.* (Child, 11b)

*I did tell some of [my friends] but not all of them.* (Child, 15a)

*The people at home they know, and the lady who is my next-door neighbour, she is the one person who knew [my child] had TB.* (Caregiver, 7a)

Some respondents felt community disclosure to be more problematic than disclosure in school.

*It’s only [in our community], the complaint is only here by us but at school I didn’t get complaints from her.* (Caregiver, 11d)

*In my class they wrote a letter telling me to take my pills when the doctor gives them to me. [But I did not tell anyone else] because they are going to talk about me here in the street, and that would hurt my feelings if they talk about me.* (Child, 14a)

A minority expressed liberal attitudes towards open disclosure.

*You know, my community [has disclosure issues]. But I am very open-minded, and my family is very open-minded, so as a result, it didn’t bother us much [that everyone knew].* (Caregiver, 17b)

*Yes I told [my friends]. […] [They] would ask me why I’m taking my medication, I would then tell them that I have TB, they then said ok. […] They were the same [after TB], nothing changed.* (Child, 17c)

One parent reasoned it was imperative to discuss children’s illnesses among friends, “*because we were a group*,” (Caregiver, 9b) suggesting a collective responsibility to protect the community from disease. Anecdotes of inadvertent, secondary disclosure were not uncommon, particularly as a consequence of clinic visits.

*The neighbours know. They asked because they could see her going to the clinic all the time.* (Caregiver, 7b)

*You come to the day hospital, all the people are sitting together, now everyone can see you are coming there to wear a mask. It’s not private, all those people know your business now.* (Caregiver, 11a)

*They see we go [to the clinic] and then they want to know where we are going, and the people see you walking over the fields… They know.* (Caregiver, 11d)

*In the mornings I would be late for class if I had to go to the clinic first. […] I also did not want to go in the afternoons because then everyone would be at the clinic.* (Child, 17a)

Still other respondents avoided disclosure entirely, choosing to keep such knowledge private.

*It’s very hard for me to tell some of [my] friends, because at the end of the day they will talk things to him.* (Caregiver, 14a)

*I really do not feel comfortable discussing it with kids my own age because you cannot predict what they might do. They could tell other people then I would look like the bad one, and as soon as people who do not have TB start talking about TB, like in my class for example, then I would feel uncomfortable and guilty.* (Child, 17a)

In the words of one concerned mother, “*Here in these flats, you can’t get sick.*” (Caregiver, 11d) The caregiver did not elaborate her statement further, suggesting only that gossip was a serious problem in her community.

#### Stigma and fear of infection

Public stigma and a fear of being infected with MDR-TB were frequently cited as drivers of respondents’ reluctance to discuss TB status. However, only a few participants reported experiencing enacted stigma.

*She always says that the kids tease her sometimes […] [They call her] “half lung” and “you sick” and so on.* (Caregiver, 8)

*[The neighbourhood children] call me names and they hit me and stuff like that. [They would say] ‘TB thing, just go away.’ They think I’m going to infect them.* (Child, 12)

Generally, social distancing was limited to the child perceived to be infectious. Lack of education about TB infectivity was cited as the underlying cause of stigma.

*[Some] children don’t understand. And their parents don’t understand. Because everybody knows about this sickness. Sometimes, years before the treatment, the people were scared somebody [would] come next to them.* (Caregiver, 10)

*We live in a community that’s not very educated about anything here. So it’s like, I keep my child here and you keep your child there.* (Caregiver, 11a)

One parent challenged this stigma by educating her community about TB treatment.

*I told [parents in the community] that it is not dangerous because she is on treatment. Yes, I even told them their cousins stayed with them so there is nothing wrong, they live in the same house eating the same food with the same dishes and spoons.* (Caregiver, 9a)

Another parent shared similar information with her child’s peers.

*I talked to [her friends] and I said, “You don’t get TB when you are on treatment, if you are off treatment [or] you don’t take your treatment, then you get TB.”* (Caregiver, 11d)

Promisingly, few respondents cited persisting stigma once it was clear that children were no longer infectious.

*Before she even finished treatment, everything was back to normal.* (Caregiver, 11a)

*I talked to [my friends] and then when I coughed, they would just say, ‘Keep your hand in front of your mouth.’ They’re like normal [now].* (Child, 11d)

Among children who disclosed their TB status to their peers, only one reported still experiencing stigma after TB treatment.

#### Relationships within the family

The impact of treatment on family relationships was varied, ranging from the dissolution of marriage to an increase in family intimacy.

*[My husband and I] argued because I stayed at the hospital for a very long time and he felt like I cared more about the child than I did for him. Then he left, and then everything was my responsibility.* (Caregiver, 15a)

*You could really see that [my children] grew closer together, in fact the whole family at the time.* (Caregiver, 17b)

Another caregiver refused to answer questions about impact on family relationships.

*I don’t want to talk about these things, really I don’t want to talk.* (Caregiver, 11d)

Family support was also evident, particularly with respect to fulfilling the demands of childcare.

*[The child’s mother] was very sick, she also had TB and she slept a lot and did not have the strength to get up and take care of the kids, so then my other sister helped them out a lot.* (Caregiver, 8)

*The whole family was very supportive; they supported me every time I must go to the hospital, they ask, “Can I take you?” and the one can go to work or so.* (Caregiver, 9b)

*My daughter would come [visit her sister in hospital] every evening from work, my son was studying at the time but he would also [visit her]. (Caregiver, 17b)*

Overall, respondents whose stories evoked the involvement of multiple family members during the child’s treatment period appeared better able to share the burden of illness relative to caregivers without such support.

#### Health costs to family members

Although self-reported adverse psychological effects among caregivers were relatively infrequent, these were particularly acute among those who had sustained previous trauma.

*When [my daughter] became ill, it seemed like I was also going down […]. It felt like my body just couldn’t take it anymore, because my [other] baby passed on because she had diarrhoea […]. Everything was too much for me. (Caregiver, 11c)*

*I was very sad because I lost a daughter to leukaemia, and now my husband got extremely drug-resistant TB [XDR-TB], and my baby got TB, and a lot of things go wrong.* (Caregiver, 11d)

An elderly caregiver reported similar psychological stress and ensuing adverse health effects.

*I was very upset, I didn’t feel right, I got sick myself. […] I’m diabetic and have high blood pressure. I was worse by the time she was sick.* (Caregiver, 9a)

For some, caring for a chronically ill child was accompanied by feelings of guilt.

*There were times that the [four] kids [at home] had to sleep alone, and the father was working and I had to ask other people to look after them, so I couldn’t be there for them. I was torn up. (Caregiver, 11a)*

In spite of their own struggles however, caregivers frequently prioritised the child’s wellbeing above all else.

*I said [to my son], “Yes, I will visit you [in hospital] because I love you, ever since I am a mother for you. I make everything for you, don’t worry.”* (Caregiver, 14a)

*I did everything possible for [my daughter]. […] They would give me the medication and I would administer. I would bathe her, all of that.* (Caregiver, 17b)

#### Mitigating effect of social support

Children admitted to hospital were allowed to return home every second weekend, so as to minimise interference with the injection schedule, and appeared to draw great comfort from their continued inclusion in familiar social networks.

*After a month, [the hospital] gave [my child] to me on weekends, and they also taught me the right way of giving her pills.* (Caregiver, 9a)

*Every second weekend [my friends came to see me] when I came home from hospital.* (Child, 12)

*[Her friends at school] missed her, the children made cards for her.* (Caregiver, 12)

*On my 16*^*th*^*birthday my aunt and her church people came and they also brought along some sweets for me. […] I was so happy to see them because I never thought that they would visit me.* (Child, 17a)

Furthermore, internalised stigma was reduced when children learned of individuals whose social status challenged the negative associations of people who develop TB disease.

*I said to [my brother] “you are not shy [about taking TB treatment]!” The girls go crazy over him. He would then ask why he should be shy, because there are a lot of gangsters who are sick who go to the clinic as well. So then when he takes his tablets I would take my tablets as well.* (Child, 17a)

Teachers took on supportive roles for both children and caregivers, including helping to monitor the child at school and administer treatment.

*I had to involve the teacher in school as well, and then she would drink [her pills] with the teacher […]. So me and the teacher had to communicate with each other on a regular basis.* (Caregiver, 11a)

*Oh the school, they really did support us, all the way.* (Caregiver, 11d)

*[T]he teacher phoned her twice a week just to find out how she’s doing.* (Caregiver, 12)

*[The children in school] started making fun of [my child] and the teacher told her, “Just ignore them.” Afterwards she had a chat with [the children] and told them what MDR-TB is all about.* (Caregiver, 12)

*My teacher phoned [the hospital] from his classroom. I cried on that day because I missed my classmates and I missed everyone at home.* (Child, 17a)

Teachers’ attentiveness to children and caregivers’ needs was conducive to both emotional and social rehabilitation, as well as to facilitating the management of at-home care.

## Discussion

This exploratory study revealed that children’s experiences of MDR-TB treatment were heterogeneous. Importantly, the key findings emerging from the present study echo the stories of children affected by MDR-TB worldwide. A recently published collection of 30 stories of children affected by MDR-TB from 30 different countries revealed the critical need for a comprehensive approach to MDR-TB treatment [8]. In addition to the urgent need for improved diagnostics and child-friendly TB drug development, these stories demonstrate that psychosocial support interventions including pain management, hearing tests, nutritional support, and travel allowances are equally instrumental to positive health outcomes for children and their families.

Findings from the current study confirmed what is known about TB treatment: pills are unpalatable and excessive in number, and adverse effects are highly disruptive, producing little incentive for children to remain adherent to treatment [7, 20]. Furthermore, medications frequently produce acute academic disruptions, sometimes persisting beyond the termination of treatment. In particular, some children may be at higher risk of experiencing cognitive treatment-related adverse effects, including various psychiatric disorders [21, 22]. These may in turn have consequences for the child’s long-term academic and social reintegration. Brief psychosocial screenings would help identify those children at risk prior to the start of MDR-TB treatment.

Similarly, assessments of caregivers could also identify individuals at risk of experiencing pathological stress, anxiety, or depression. While most respondents did not report changes in family relationships, a minority highlighted acute distress, including spousal separation. Research has shown that caregivers may experience high levels of disability-related stress resulting from the strain of dealing with a chronically ill child [23]. This was evident in caregivers’ accounts of their own physical and mental health struggles, which were frequently aggravated during the child’s illness. In turn, children’s psychological vulnerability is especially pronounced when parents suffer from poor health or emotional distress [24]. As caregivers almost always accompany children to medical appointments, health professionals could use this setting to assess the presence of stressors which may impact on caregivers’ well-being and management of childhood illness [25].

Virtually all caregivers reported financial difficulties resulting from treatment-related expenditures. Despite financial distress however, most caregivers paid regular visits to children in hospital. Currently, all TB treatment (including treatment for MDR-TB) and hospitalisation is free of charge for all public sector patients in South Africa. In addition, government grants offer child support payments (Totalling R280 [USD 26] per month in 2012) to couples earning a combined salary of less than R5,600 (USD 524) per month, or to single parents with a salary of less than R2,800 (USD 263) per month [26]. This grant is available to parents for each child under 18 years of age. If a child is unwell, however, family expenditures can exceed support payments, particularly if caregivers are unemployed. Caregivers who qualify for grants are informed by social workers at both Tygerberg and Brooklyn Chest hospitals; however, it is unclear what proportion of respondents in this study had access to grant payments, or whether any remained unaware of their existence.

In agreement with our findings, a recent systematic review on the pathways of TB stigma revealed the most common cause of stigma to be the perceived risk of transmission to vulnerable individuals [27]. While knowledge interventions are especially effective against such instrumental stigma [28], interventions must simultaneously guard against inadvertently contributing to an increased fear of infection due to the prevalence of self-protection messages [29]. Given its protective function, education interventions could reduce instrumental stigma, but are unlikely to eliminate it altogether. Interventions should therefore aim to target stigma that is unjustified in terms of self-protection, including the avoidance of a child who is no longer clinically infectious.

With the exception of a few adolescents who displayed signs of internalised TB stigma, most children did not appear to suffer lasting adverse effects. Importantly, children who did not internalise stigma also tended to experience strong community, family, and peer support. Some evidence suggests that involving role models in TB discourse could further guard against self-stigma, for instance, by elevating respected public figures who survived TB disease. Research originating from the Eastern Cape revealed that few citizens were aware such figures included Nelson Mandela and Desmond Tutu [30]. In addition, a recent study found that schoolchildren in high-burden TB settings were key players in the broader communication of TB information [31]. Schools should therefore encourage classroom discussions of TB stigma, its consequences, and provide resources to which children could turn for psychosocial support.

This study has several potential limitations. Time constraints restricted the sampling strategy to participant availability. The use of an interpreter may have resulted in information omissions and misinterpretations. Additional limitations included the use of caregiver proxy interviews to substantiate children’s experiences. All participants may have been subject to interviewer and recall bias, with the potential for additional bias resulting from the demand characteristics of the research setting: children’s desire to please may sometimes outweigh their desire to be truthful. Lastly, the interviewer’s association with a research organisation could have reduced participants’ willingness to express criticism towards health care workers or medical treatment.

## Conclusions

The current treatment regimen for MDR-TB is problematic in terms of its duration, complexity, and the adverse events associated with medications. Although there appear to be no long-lasting effects in younger children, they, like all children, would strongly benefit from a shorter, more manageable treatment regimen. Finally, there is a need for psychosocial support of both children and caregivers to mitigate the potential negative effects of stigma, and help them manage difficulties related to disclosure. Multi-level interventions should aim to provide a framework of support for children and their families, beginning at the time of diagnosis and continuing into the child’s outpatient treatment.

## Electronic supplementary material

Additional file 1: Inclusion of eligible participants.(DOC 64 KB)
