# Supplementary material for: Assessing the impact of multidrug-resistant tuberculosis in children: an exploratory qualitative study
Source: BMC Infect Dis. 2014 Aug 1;14:426. doi: 10.1186/1471-2334-14-426 (PMC4127187; doi:10.1186/1471-2334-14-426)
Supplement: Supplementary file 1 — Additional file 1: Inclusion of eligible participants.(DOC 64 KB) [file 12879_2014_3724_MOESM1_ESM.doc]

Additional file 1. Inclusion of eligible participants.

Children referred to TCH & Khayelitsha outreach clinics, started on treatment for MDR-TB between 1^st^ January, 2009 and 1^st^ January, 2010

(n = 148)

Excluded (n = 107):

- Did not meet age eligibility criteria

(n = 107)

Children at least 5 years of age at the start of MDR-TB treatment

(n = 41)

Excluded (n = 5):

- Resides >1 hr drive from research

facilities (n = 3)

- Deceased (n = 2)

Children eligible for purposive sampling

(n = 36)

Abbreviations: TCH = Tygerberg Children’s Hospital
